# Ovarian germline stem cell dedifferentiation is cytoneme dependent

**DOI:** 10.1073/pnas.2426145122

**Published:** 2025-08-26

**Authors:** Catherine Sutcliffe, Nabarun Nandy, Raluca Revici, Heather Johnson, Shukry J. Habib, Hilary L. Ashe, Scott G. Wilcockson

**Affiliations:** ^a^School of Biological Sciences, Faculty of Biology, Medicine and Health, University of Manchester, Manchester M13 9PT, United Kingdom; ^b^Department of Biomedical Sciences, Lausanne 1005, Switzerland

**Keywords:** germline stem cell, cytoneme, BMP signaling, dedifferentiation, enabled

## Abstract

Progenitor cell dedifferentiation is important for stem cell maintenance during tissue repair and age-related stem cell decline. Here, we use the *Drosophila* ovary as a model to study the role of cytonemes in bone morphogenic protein (BMP) signaling–directed germline stem cell (GSC) maintenance and dedifferentiation of germ cells to GSCs. We provide evidence that differentiating germ cell cysts extend longer cytonemes that are more polarized toward the niche during dedifferentiation to reactivate BMP signaling. The presence of additional somatic cells in the niche is associated with a failure of germ cell dedifferentiation, consistent with the formation of a physical barrier to cytoneme–niche contact and outcompetition of germ cells for BMP. Using BMP beads in vitro, we show that these are sufficient to induce cytoneme-dependent contacts in *Drosophila* tissue culture cells. We demonstrate that the Enabled (Ena) actin polymerase is localized to the tips of germ cell cytonemes and is necessary for robust cytoneme formation, as its mislocalization reduces the frequency, length, and directionality of cytonemes. During homeostasis, specifically perturbing cytoneme function through Ena mislocalization impairs GSC fitness by reducing GSC BMP signaling and niche occupancy. Disrupting cytonemes by targeting Ena during dedifferentiation reduces germ cell BMP responsiveness and the ability of differentiating cysts to dedifferentiate. Overall, our results provide evidence that cytonemes play a fundamental role in establishing polarized signaling and niche occupancy during stem cell maintenance and dedifferentiation.

## Significance

Fertility depends not only on germline stem cell (GSC) maintenance during homeostasis but also on the ability of germ cells to reverse their developmental program and dedifferentiate during aging- or stress-induced GSC loss. Here, using the *Drosophila* ovary as a model, we show that cytonemes are required for GSC asymmetric signaling, niche occupancy, and fitness during homeostasis. During dedifferentiation, we demonstrate that cytonemes are necessary for differentiating germ cells to reaccess the self-renewal signal and establish polarized signaling, recolonize an empty niche, and dedifferentiate to germline stem cells. Our data identify cytonemes as a target for strategies aimed at improving stem cell fitness or dedifferentiation, which has huge therapeutic potential for the regeneration of damaged tissues.

Stem cells reside in a niche, a specialized microenvironment that provides physical support and signals to control stem cell maintenance (1). Actin-rich signaling filopodia called cytonemes (2, 3) mediate stem cell–niche communication in different contexts. For example, *Drosophila* hematopoietic niche cell cytonemes deliver Hedgehog (Hh) signals to blood progenitor cells to maintain their fate. Cytonemes are disrupted upon infection, resulting in ectopic differentiation of progenitors into hemocytes to strengthen the immune response (4). *Drosophila* adult muscle progenitors (AMPs) extend cytonemes loaded with fibroblast growth factor (FGF) receptors to adhere to wing disc niche cells and receive the FGF signal, resulting in asymmetric signaling and AMP-niche organization. There are two spatially restricted niches: Each expresses a different FGF ligand and supports a distinct population of muscle-specific AMPs due to specific cytoneme–niche contacts (5). Embryonic stem cells (ESCs) extend cytonemes carrying Wnt receptors to pair with cocultured trophoblast stem cells (TSCs), receive the self-renewal Wnt signal, and initiate formation of embryo-like structures (6). In contrast, the cytonemes from ESCs that have transitioned to a primed state are unable to form stable interactions with Wnt sources, impairing TSC pairing and synthetic embryogenesis (7).

**Fig. 1.**
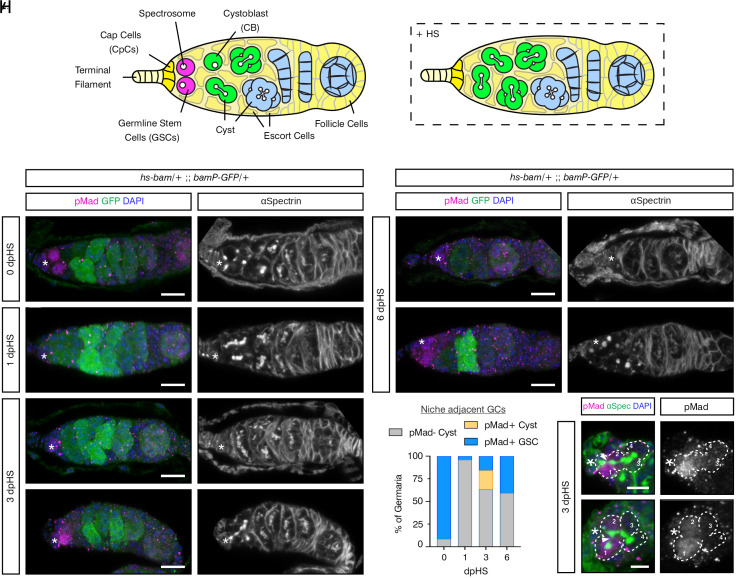
GCs dedifferentiate over time to reoccupy the niche. (*A*) Schematic of the germarium with the different cell types, as labeled, anterior is to the *Left*. The spectrosome and fusomes are shown in white. The boxed germarium shows post-HS induced *bam* expression. (*B*–*G*) Germaria carrying *bam* promoter-GFP and *hs-bam* transgenes immunostained with anti-pMad (magenta), anti-GFP (green), anti-αSpectrin (gray, *Right* panel), and DAPI (blue). Representative germaria are shown for day 0 (*B*) and 1 dpHS (*C*), 3 dpHS (*D* and *E*), and 6 dpHS (*F* and *G*). (*) Cap cells. (Scale bar, 10 μm.) (*H*) Quantitation of the number of germaria where the GCs contacting the niche are pMad-positive or -negative differentiating cysts or pMad-positive GSCs on 0, 1, 3, and 6 dpHS. The number of germaria counted per time point was 104, 100, 101, and 103 for 0, 1, 3, and 6 dpHS, respectively. (*I* and *J*) pMad-positive cysts at 3 dpHS immunostained with anti-pMad (magenta, gray *Right* panel), anti-αSpectrin (green), and DAPI (blue). The image in (*I*) is from the germarium shown in (*E*). Connected CBs are outlined and numbered 1 to 4 in the 4-cell cyst. (*) Cap cells, the arrowhead shows a fusome undergoing scission. (Scale bar, 5 μm.)

*Drosophila* ovarian germline stem cells (GSCs) also use cytonemes for short-range niche communication. The GSC niche is composed of specialized somatic cells, including cap cells (CpCs) and escort cells (ECs), which provide self-renewal signals to support 2 to 3 GSCs per germarium (8, 9). GSCs undergo asymmetric division to form one GSC, which remains in the niche, and one daughter cystoblast (CB) which leaves the niche and differentiates. The key signal promoting stem cell identity is Bone Morphogenic Protein (BMP) signaling, driven primarily through the ligands Decapentaplegic (Dpp) and Glass Bottom Boat (Gbb), resulting in phosphorylation of the transcription factor Mothers against Dpp (Mad) (10, 11). In addition, GSCs are anchored to the CpCs through E-Cadherin (Ecad) mediated cell adhesion at adherens junctions (12). GSCs are therefore identifiable by their anterior localization, high pMad levels, and the presence of the spectrosome, a germline-specific spectrin-rich endomembrane organelle that becomes a branched structure (fusome) in more developed cysts (Fig. 1*A*). BMP signaling silences transcription of the differentiation master gene, *bag of marbles* (*bam*) (11, 13). Upon GSC division, the daughter cell that exits the niche has reduced pMad and upregulates *bam* expression, triggering germline differentiation to a CB (14, 15). A single CB undergoes four rounds of mitosis with incomplete cytokinesis to form 2-, 4-, 8-, and 16-cell cysts (cc) with interconnected fusomes (16, 17).

Restricted access to BMP is an important regulatory step that limits stem cell identity to those cells in direct contact with the niche (18). GSC–niche signaling is mediated in part by cytonemes (19). It has been shown that early germ cells (GCs) form actin-rich projections and GSCs extend cytonemes to probe the niche and access Dpp bound by the heparan sulfate proteoglycan Division abnormally delayed (Dally) to promote BMP signal transduction. Dpp signaling induces the formation of more stable microtubule-enriched cytocensors, which also receive the BMP signal but provide feedback inhibition through signal suppression (19). Short cytonemes mediating Hh distribution have also been detected in the germline niche emanating from Hh-producing CpCs toward receiving ECs (20). Thus, cytonemes play an integral role in cell communication in the ovarian germline.

Many factors, particularly those associated with the actin cytoskeleton, have been identified as regulators of cytoneme formation (3). Enabled (Ena), an actin polymerase, is associated with filopodia extension in many cellular contexts (21–24). Ena localizes to the tips of lamellipodia and filopodia (24–27), where it recruits Profilin/actin complexes to enhance F-actin polymerization (28, 29) and filopodia formation (30, 31). In addition, Ena localizes to focal adhesions and adherens junctions (32). In the latter, Ena plays a role in Ecad localization (33) and in cytoskeletal regulation at Ecad contact sites (32). Ena is therefore an attractive candidate that could regulate GSC cytoneme formation and niche interaction.

It has previously been shown that transient induction of *bam* expression using a heat shock (HS)-inducible transgene, *hs-bam*, causes complete loss of GSCs from the niche through Bam-induced differentiation within 24 h (34). Following this, over the course of a few days, GCs can recolonize the niche and fully recover GSC loss. This is driven by cyst dedifferentiation, resulting in gradual cyst breakdown to form single cells (35). Here, we used this system to test the role of cytonemes in mediating BMP-dependent dedifferentiation. We show that Ena loss-of-function decreases the robustness of cytoneme formation. This reduces GC responsiveness to BMP signaling and niche occupancy, which impairs GSC fitness during homeostasis and precludes niche recolonization during GC dedifferentiation.

## Results

### Early Cysts Can Dedifferentiate to Recolonize an Empty GSC Niche.

To investigate a role for cytonemes in GSC dedifferentiation, we first established the timeline of BMP responsiveness and dedifferentiation using a previously described assay (35, 36). We transiently induced expression of the differentiation factor *bam* throughout the germarium using an *hs-bam* transgene. The number of pMad-positive early GCs was visualized in the days post-HS (dpHS) to quantify the level of dedifferentiation and recovery of niche-associated GSCs. The presence of pMad indicates that the cells are responding to niche-derived Dpp signaling. A transgene with the *bam* promoter driving GFP (*bamP-GFP*) (13) was also used to mark Bam-positive differentiating cells. Pre-HS, pMad-positive GSCs are identified by the presence of an anteriorly localized spectrosome (visualized by αSpectrin staining) associated with niche CpCs (Fig. 1 *A* and *B*). GFP is detected in differentiating CBs and developing cysts located more posteriorly (Fig. 1*B*).

One dpHS, pMad-, and spectrosome-positive GSCs are absent, and the cells directly adjacent to the niche are GFP-positive with branched fusomes (Fig. 1*C*). This indicates that GSCs and early CBs have been lost due to differentiation (Fig. 1*A*). Indeed, almost all germaria contain pMad-negative cysts (Fig. 1*H*; 1 dpHS cf day 0 pre-HS). At 3 dpHS, while ~60% of germaria still only contain differentiated cells (Fig. 1*D*), ~40% now contain pMad-positive and GFP-negative cells adjacent to the niche, consistent with dedifferentiation (Fig. 1*E*). pMad accumulation is either associated with a spectrosome-containing GSC in direct contact with the niche or a cyst containing a branched fusome (Fig. 1*H*). Within these pMad-positive cysts (typically at the 4 cc stage) only the cells in direct contact with the CpCs, the source of Dpp, are pMad-positive (Fig. 1 *I* and *J*). Fusome thinning and scission, which are associated with cyst breakdown during dedifferentiation (35), are also evident at the 4 cc stage.

By 6 dpHS, the majority of germaria continue to display a lack of dedifferentiation such that all early GCs are lost, and only later-stage cysts are present (Fig. 1 *F* and *H*). This suggests that after 3 dpHS, there is no further dedifferentiation of cysts. However, in germaria where dedifferentiation has occurred, pMad- and spectrosome-positive GSCs are detected in the niche (Fig. 1*G*). The proportion of germaria with pMad-positive GSCs that have dedifferentiated is highest at 6 dpHS (Fig. 1*H*). Together, these data show that this post-HS time course can capture different events during GC dedifferentiation, including the ability of cysts to respond to Dpp signaling, followed by cyst breakdown and recolonization of the GSC niche.

### Dedifferentiating GCs Extend Cytonemes during Niche Recolonization.

**Fig. 2.**
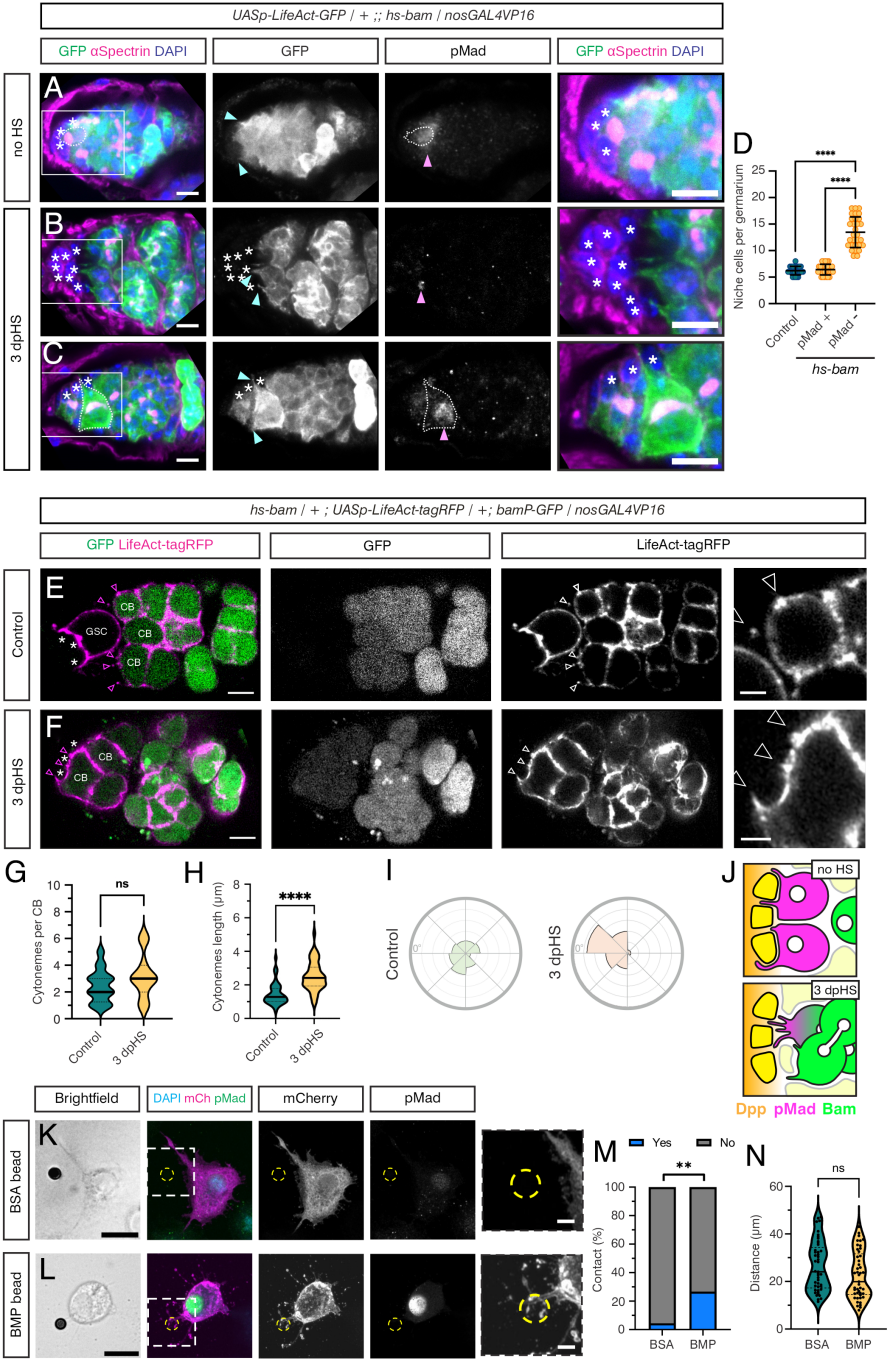
Dedifferentiating GCs extend cytonemes during niche recolonization. (*A*–*C*) Germaria carrying *UASp-LifeAct-GFP* driven by *nosGAL4VP16* and an *hs-bam* transgene immunostained with anti-GFP (green and *Inset*), anti-αSpectrin (magenta), anti-pMad (*Inset*), and DAPI (blue). The right panels show higher magnification views of the boxed areas. Non-HS control (*A*), 3 dpHS germaria (*B* and *C*) with (*B*) a pMad negative 8 cc extending cytonemes to the niche and (*C*) a pMad-positive GSC with cytonemes contacting the niche. (*) Niche cells including extra somatic cells, cyan arrowheads mark cytonemes, magenta arrowheads indicate pMad-positive cells, white dotted lines outline pMad-positive GSCs. (Scale bars, 5 μm.) (*D*) Graph shows quantitation of the number of niche cells in *hs-bam* germaria (3 dpHS) that contain pMad-positive dedifferentiating GCs or only pMad-negative GCs, compared to control germaria (HS, with no *hs-bam* transgene). n = 30, data shown as mean ± SD, ANOVA *P* =< 0.001 (control vs. pMad− GCs), *P* =< 0.001 (pMad+ GCs vs. pMad− GCs). (*E* and *F*) Images of live germaria carrying *UASp-LifeAct-tagRFP* driven by *nosGAL4VP16* (magenta and *Inset*), a *bamP-GFP* reporter to mark differentiating cells (green and *Inset*), and an *hs-bam* transgene. Non-HS control (*E*) and 3 dpHS germaria (*F*) show the cytonemes on a GSC and CBs (*E*, arrowheads) or a CB occupying the niche (*F*, arrowheads). Higher magnification views of *LifeAct-tagRFP* labeled CB cytonemes are shown in the *Right* panel. (*) Niche cells. (Scale bar, 5 μm and *Inset* 2μm.) (*G*–*I*) Quantification of the number of cytonemes per CB (*G*), cytoneme length (*H*), and orientation relative to the niche (0°), each interval is 10% (*I*). n = 50 (*hs-bam*) and 46 (control), Welch’s unpaired two-tailed *t* test, *P* < 0.0001, ns not significant. (*J*) Schematic of the germarium niche showing no HS GSCs and a 3 dpHS cyst which extends cytonemes toward the niche and is BMP responsive. (*K* and *L*) S2R+ cells transfected with Tkv-mCherry and FLAG-Mad expression plasmids and incubated with BSA (*K*) and BMP2 (*L*) coated beads. S2R+ cells were immunostained with anti-tdTomato (magenta and *Inset*) and anti-pMad (green and *Inset*). Bead locations are marked with yellow dashed lines. Brightfield images and higher magnification views of the Tkv-mCherry channel are shown. (Scale bar, 10 μm and *Inset* 2μm.) (*M*) Quantification of the contacts between BSA and BMP2-coated beads and S2R+ cells (examples are shown in *K* and *L*). n = 45, Fisher’s exact test, *P* = 0.0071. (*N*) Quantification of the distance between the bead and the center of the cell nucleus. Mann–Whitney *P* = 0.0781.

As we previously showed that cytonemes enable GSCs to access niche Dpp (19), we used the dedifferentiation assay to test whether cytonemes play a role in the dedifferentiation of early cysts by enabling access to niche-derived Dpp. To visualize cytonemes during dedifferentiation, we used the germline-specific *nosGAL4VP16* driver to activate a *UASp-LifeAct-GFP* reporter and observed GCs 3 and 4 dpHS. Pre-HS control germaria were also studied, which contain GSCs with niche-directed cytonemes (Fig. 2*A*).

Post-HS, germaria in various stages of dedifferentiation were identified. Some germaria have only early or late cysts remaining and lack cytonemes (*SI Appendix*, Fig. S1 *A* and *B*). In contrast, other germaria show pMad-negative early cyst cells extending cytonemes toward the niche (Fig. 2*B* and *SI Appendix*, Fig. S1*D*). We also observed germaria containing cysts with a low pMad level and short, niche-directed cytonemes (*SI Appendix*, Fig. S1*C*), as well as germaria in which GSCs have recolonized the niche (Fig. 2*C* and *SI Appendix*, Fig. S1*E*). These pMad-positive GSCs have cytonemes contacting the niche (Fig. 2*C* and *SI Appendix*, Fig. S1*E*), as observed for non-HS controls (Fig. 2*A*).

In some germaria with cysts, extra somatic cells are found to accumulate in the niche (Fig. 2*B* and *SI Appendix*, Fig. S1 *A*, *B*, and *G*), some of which are pMad-positive (Fig. 2*B*, pink arrowhead), indicating that they are receiving BMP signaling. This is consistent with a previous report of somatic ECs and follicle cell progenitors populating the niche after GSC loss following *hs-bam* expression (36). We hypothesized that accumulation of nongermline cells in the niche could block access for the differentiating cysts preventing them from reoccupying the niche. To test this, we quantitated both the number of niche cells and pMad-positive GCs in germaria undergoing dedifferentiation following an *hs-bam* pulse (*SI Appendix*, Fig. S1 *F*–*K*). These data show that *hs-bam* germaria with pMad-positive GCs have the same number of niche cells as in control germaria, whereas germaria with extra niche cells contain only pMad-negative GCs (Fig. 2*D*). This dichotomy supports the idea that extra cells in the niche act as a barrier to dedifferentiation.

To study the cytonemes in more detail, we quantitated the number, length, and orientation in CBs in the process of niche recolonization in 3 dpHS *hs-bam* germaria, compared to CBs in control non-HS germaria. These germaria also have germline expression of *LifeAct-tagRFP* and carry the *bamP-GFP* reporter that marks differentiating cells (Fig. 2 *E* and *F*). The quantitation shows that the dedifferentiating CBs have the same number of cytonemes as the control CBs from non-HS germaria (Fig. 2*G*). However, the cytonemes on dedifferentiating CBs are longer and more niche oriented (Fig. 2 *H* and *I*). To measure cytoneme orientation, a line was drawn from the center of each GSC toward the niche, with this position marked as 0° on the plots (Fig. 2*I*). This increased length and niche orientation may reflect the increased space available during dedifferentiation when GSCs are lost from the niche. Together, these data show that post-HS, when the niche is empty, the cysts begin to extend cytonemes toward the niche. Over time, if the cysts are not blocked by the accumulation of somatic cells in the niche, they are able to access Dpp, reactivate pMad, and dedifferentiate as GSCs (Fig. 2*J*).

The cytonemes on dedifferentiating cells are oriented toward the BMP-expressing niche (Fig. 2*I*), consistent with previous reports of cytonemes polarized toward BMP sources in the wing disc (37–39). Therefore, we took a reductionist approach to determine whether a Dpp source is sufficient to induce cytoneme contacts. To this end, we used *Drosophila* S2R+ cells and beads that were either coated with BMP2 or BSA as a negative control. S2 cells have previously been used as a model to study Hedgehog-containing cytonemes (40). First, we tested whether the BMP2 beads were bioactive based on their ability to induce pSmad1/5 activation when added directly to C2C12 cells. These data show that the BMP2 beads activate pSmad1/5, albeit in a lower proportion of cells than observed with soluble BMP2, whereas there is no pSmad1/5 activation in either the control or BSA bead-treated cells (*SI Appendix*, Fig. S2 *A*–*C*).

Next, we tested the ability of BMP2 or control BSA beads to induce cytoneme contacts from S2R+ cells that are transfected with expression plasmids encoding the BMP receptor Thickveins (Tkv), as a Tkv-mCherry fusion, and Flag-Mad. Flag-Mad was transfected to increase the cell’s BMP responsiveness. In the analysis, we included cells that had at least one bead within a distance of 50 µm from the center of their nucleus. These data show that ~25% of S2R+ cells extend cytoneme contacts to the BMP2 beads (Fig. 2 *K* and *M*). This is significantly greater than the small proportion of contacts observed with the BSA beads (Fig. 2 *L* and *M*), despite there being no significant difference in the distances between the center of the cell nucleus and beads across the cells analyzed (Fig. 2*N*). Other examples of S2R+ cytoneme contacts with BMP2 beads and the quantitation from additional biological replicates are shown in *SI Appendix*, Fig. S2 *D*–*G*. Therefore, in this simple system, a bioactive BMP2 bead can induce contacts from Tkv-loaded cytonemes from S2R+ cells. Although this system is heterologous, the findings are broadly relevant to dedifferentiation and other cell fate outcomes by showing that a BMP source can be sufficient for cytonemes to sense and orient toward this source. Together, these in vitro and in vivo data support a model in which, upon GSC loss, the differentiating cells extend cytonemes to the ovarian niche Dpp, promoting BMP responsiveness and niche recolonization.

### Ena Is Localized at the Tips of GSC and CB Cytonemes.

**Fig. 3.**
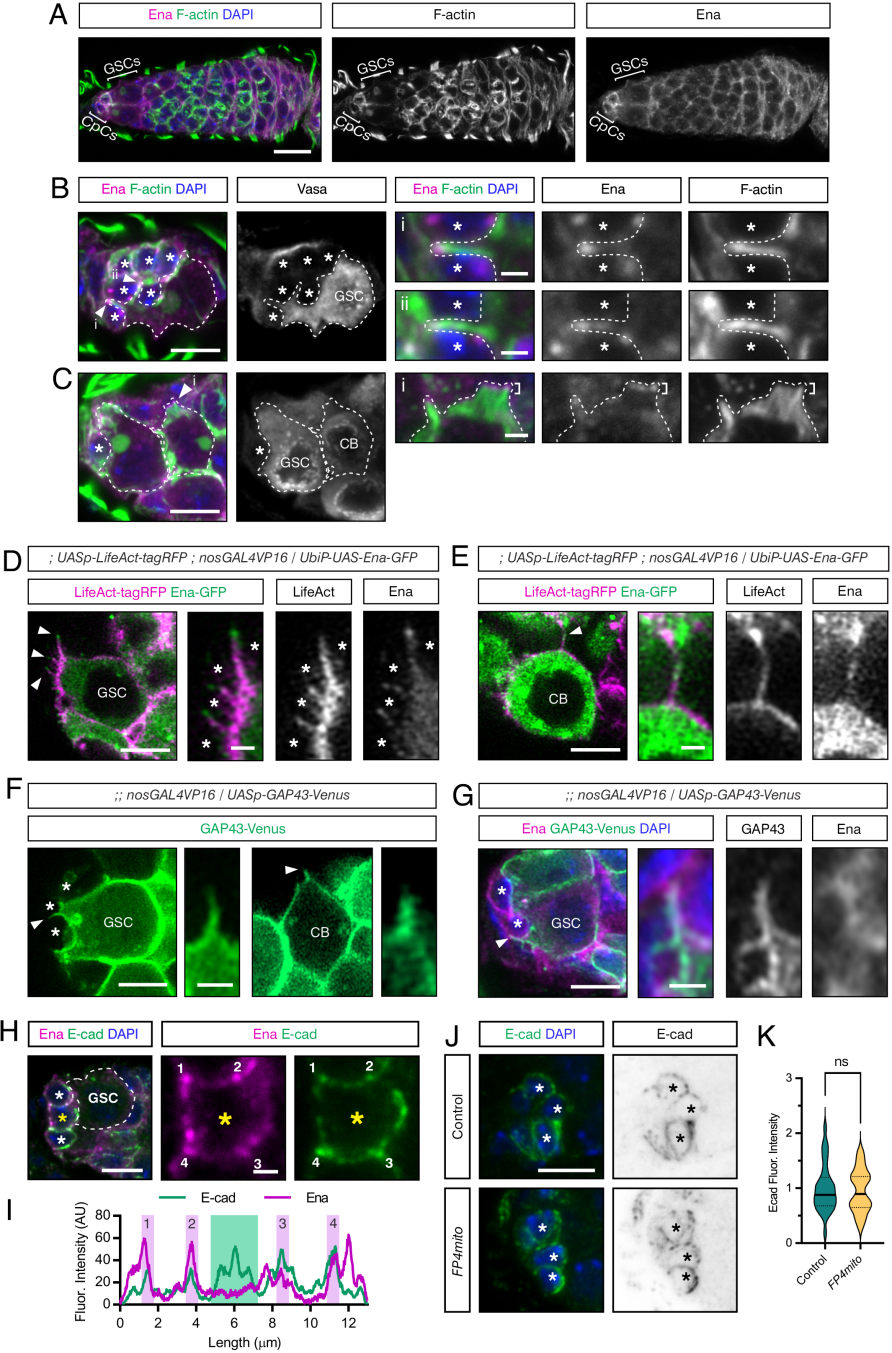
Ena is localized to cytoneme tips. (*A*) Wildtype germarium immunostained with anti-Ena (magenta), Phalloidin (green), and DAPI (blue). GSCs and CpCs are marked by brackets. (Scale bar, 10 μm.) (*B*) Immunostaining as in (*A*) and Vasa (gray *Inset*). A GSC is outlined by a dotted line based on Vasa staining, white arrowheads mark cytonemes (i and ii), that are shown as higher magnification images. (*) Cap Cells. [Scale bar, 5 μm and (i-ii) 1 μm.] (*C*) Immunostaining as in (*B*). A GSC and CB are outlined by dotted lines. A CB cytoneme is indicated by a white arrowhead and shown magnified in (i). Lamellipodial projections are indicated by brackets. (*) Cap Cells. [Scale bar, 5 μm and (i) 1 μm.] (*D* and *E*) Images of a GSC (*D*) and CB (*E*) from live germaria expressing *LifeAct-tagRFP* (magenta and *Inset*) and *Ena-GFP* (green and *Inset*) driven by *nosGAL4VP16.* Higher magnification views of cytonemes are shown. (*) Cap Cells. (Scale bar, 5 μm and *Inset* 1 μm.) (*F*) Image from a live germarium expressing *GAP43-Venus* driven by *nosGAL4VP16.* A GSC and CB are shown, with higher magnification views of the cytonemes. (*) Cap Cells. (Scale bar, 5 μm and *Inset* 1 μm.) (*G*) A GSC imaged in a fixed germarium expressing *GAP43-Venus* driven by *nosGAL4VP16* and immunostained with anti-GFP (green and *Inset*), anti-Ena (magenta and *Inset*), and DAPI (blue). The higher magnification view shows a cytoneme. (*) Cap Cells. (Scale bar, 5 μm and *Inset* 1 μm). (*H*) Wildtype germarium immunostained with anti-Ena (magenta), anti-Ecad (green), and DAPI (blue). The GSC is outlined by a dotted line. (*) Cap Cells. (Scale bar, 5 μm and *Inset* 1 μm.) (*I*) Intensity plot for Ena and Ecad staining shown in (*H*) across points 1 to 4 of the CpC marked with a yellow asterisk. (*J*) Control and *FP4mito* expressing germaria immunostained with anti-Ecad (green and gray *Inset*) and DAPI (blue). (*) Cap Cells. (Scale bar, 5 μm.) (*K*) Intensity plot for Ecad staining, as shown in (*J*), n = 29 and 23, Welch’s unpaired two-tailed *t* test, *P* = 0.5942 (ns).

To test whether cytonemes promote GSC–niche recolonization by allowing access to niche Dpp, we sought to disrupt GC cytonemes without affecting other critical actin functions. To this end, we explored Ena as a potential cytoneme regulator because it regulates filopodia emergence, without affecting cell division (24, 41). We initially visualized Ena localization in GCs. Using antibody staining in fixed germaria, we identified Ena in early GCs and at the interface between the CpCs and the GSCs (Fig. 3*A*). Using Vasa staining to mark GCs, Ena was also identified at the tips of GSC cytonemes oriented toward the niche (Fig. 3 *B*, i and ii), consistent with its role as an actin polymerase (42, 43). We also detected Ena at the tips of CB cytonemes (Fig. 3*C*) and lamellipodia-like projections (Fig. 3 *C* i, bracket), suggesting that Ena may be a general regulator of GC cytonemes.

To complement these fixed images, we used superresolution imaging (Zeiss 980, airyscan) to visualize Ena distribution on cytonemes in live ex vivo germaria carrying *Ena-GFP* and *LifeAct-tagRFP* reporters activated in the germline by *nosGAL4VP16*. The Ena-GFP transgene encodes a protein fusion driven by the *ubiquitin* promoter with upstream UAS sites. The UAS sites allow stronger expression in the presence of a driver than with the weaker *ubiquitin* promoter alone. Therefore, Ena-GFP expression is specifically enhanced in the germline in the presence of the *nosGAL4VP16* driver compared to a control lacking *nosGAL4VP16* (*SI Appendix*, Fig. S3*A*). The images from live germaria show that Ena-GFP decorates the length of GSC and CB cytonemes and is concentrated on the tips (Fig. 3 *D* and *E*). Overall, this localization suggests that Ena may be important for cytoneme extension and, therefore, a potential selective target for manipulating cytoneme behavior.

As an alternative cytoneme marker to LifeAct, we also used a *UASp-GAP43-Venus* transgene, which revealed protrusions from GSCs extending toward CpCs in a previous study (44). This fusion contains the first 20 amino acids of growth-associated protein 43 (GAP43), which targets Venus to the inner leaflet of the plasma membrane (45). Imaging live germaria expressing *GAP43-Venus* in GCs using *nosGAL4VP16* revealed the presence of cytonemes on GSCs and CBs (Fig. 3*F*). In addition, Ena staining in fixed germaria with germline expression of *UASp-GAP43-Venus* reveals that Ena is localized within the cytoneme (Fig. 3*G*). Given that GAP43-Venus is an untested marker for studying GC cytonemes, we used the data from live germaria (example in Fig. 3*F*) to quantitate cytoneme properties in GSCs and CBs. These data show that GSCs typically have more cytonemes that are longer and more polarized toward the niche than the CB cytonemes (*SI Appendix*, Fig. S3 *B* and *C*). These findings are consistent with the quantitation for the control CBs (Fig. 2 *G*–*I*) and previous quantitation of GSC and CB cytonemes (19) using *LifeAct-GFP*.

To disrupt Ena function specifically in the ovarian germline, we used an established Ena mislocalization construct, *FP4mito.* FP4mito contains a multimerized FPPPP (FP4) motif, which is bound by the N-terminal EVH1 domain of Ena with high affinity, fused to a mitochondrial outer membrane targeting signal (27). Expression of an *FP4mito* transgene in vivo mislocalizes Ena to mitochondria, which inhibits Ena function. (41, 46–48). To specifically mislocalize Ena in GCs, *FP4mito* was expressed in the germline using *nosGAL4VP16* alone or with *LifeAct-GFP* to visualize F-actin. Both the Ena-GFP reporter (*SI Appendix*, Fig. S3*D*) and F-actin (*SI Appendix*, Fig. S3*E*) are observed to accumulate in cytoplasmic aggregates, only in GCs, in *FP4mito* expressing germaria, as described previously for *FP4mito* expressing epidermal cells in the *Drosophila* embryo (41).

Ena can also play a role in cellular adhesion, and we observe Ena concentrating at both GSC–CpC and CpC–CpC tricellular junctions (Fig. 3 *H* and *I*), but we do not observe Ena with Ecad at the GSC–niche interface (Fig. 3*I*). Although the data described above show that Ena is mislocalized by germline expression of *FP4mito*, we observe no effect on Ecad levels (Fig. 3 *J* and *K*), suggesting that Ena mislocalization does not impact GSC–niche adhesion at homeostasis. Overall, these data show that *FP4mito* efficiently mislocalizes Ena in the ovarian germline and therefore provides a potential tool for the perturbation of cytoneme formation.

### Ena Regulates GC Cytoneme Formation.

**Fig. 4.**
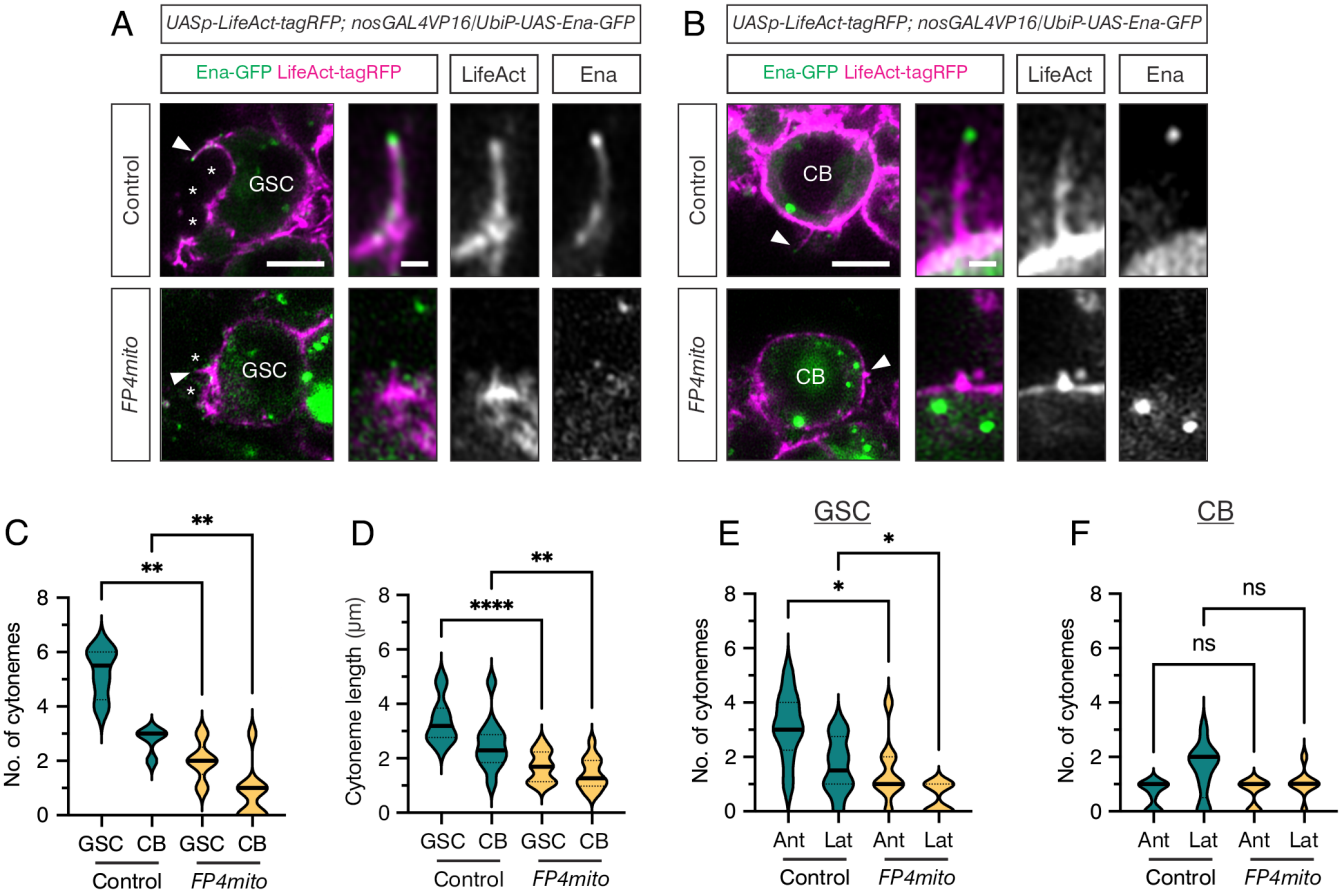
Ena regulates GC cytoneme formation. (*A* and *B*) Live images of control and *FP4mito* expressing germaria, with *LifeAct-tagRFP* (magenta and *Inset*) and *Ena-GFP* (green and *Inset*) driven by *nosGAL4VP16*. Images show a GSC (*A*) or CB (*B*), with higher magnification views of a cytoneme. (*) Cap Cells. (Scale bar, 5 μm.) (*C* and *D*) Graphs showing the number of cytonemes per GSC or CB and cytoneme lengths from live control and *FP4mito* germaria, as shown in (*A* and *B*) for representative examples. n = 11-13 cytonemes, Welch’s unpaired *t* test, ****P =< 0.0001 and **P =< 0.01. (*E* and *F*) Data from (*C* and *D*) showing the number of cytonemes per GC projecting either anteriorly or laterally from live control and *FP4mito* germaria, as shown in (*A* and *B*) for representative examples. Welch’s unpaired *t* test, *P =< 0.05.

Next, we tested the impact of *FP4mito* expression on cytoneme formation by quantifying the number, length, and orientation of GSC and CB cytonemes labeled with germline expression of *LifeAct-GFP* in live ex vivo germaria (Fig. 4 *A* and *B*). We found that when Ena is mislocalized to mitochondria, there are significantly fewer (~twofold) cytonemes on both the GSCs and CBs (Fig. 4*C*). In addition, the mean GSC and CB cytoneme length is significantly reduced by ~twofold in *FP4mito* germaria compared with controls (Fig. 4*D*). This is consistent with previous studies where mislocalization of Ena with *FP4mito* results in a reduced length of filopodia in leading edge cells during *Drosophila* dorsal closure (41).

We next characterized the directionality of GSC cytonemes. Briefly, a line was drawn from the center of each GSC toward the niche. Cytonemes within a 45° angle from this line were counted as being anteriorly localized, while cytonemes oriented more than 45° were assigned as lateral (19). *FP4mito*-expressing GSCs display a significant shift from anteriorly localized to more lateral cytonemes than controls (Fig. 4*E*), indicating that the directionality of cytoneme formation is perturbed. However, there is no significant change in the orientation of CB cytonemes in *FP4mito* germaria (Fig. 4*F*). In conclusion, Ena mislocalization perturbs GSC and CB cytoneme formation and those that are formed are shorter and, in the case of GSCs, misoriented relative to the niche.

### Ena Mislocalization Impairs BMP Signal Reception and Stem Cell Fitness.

**Fig. 5.**
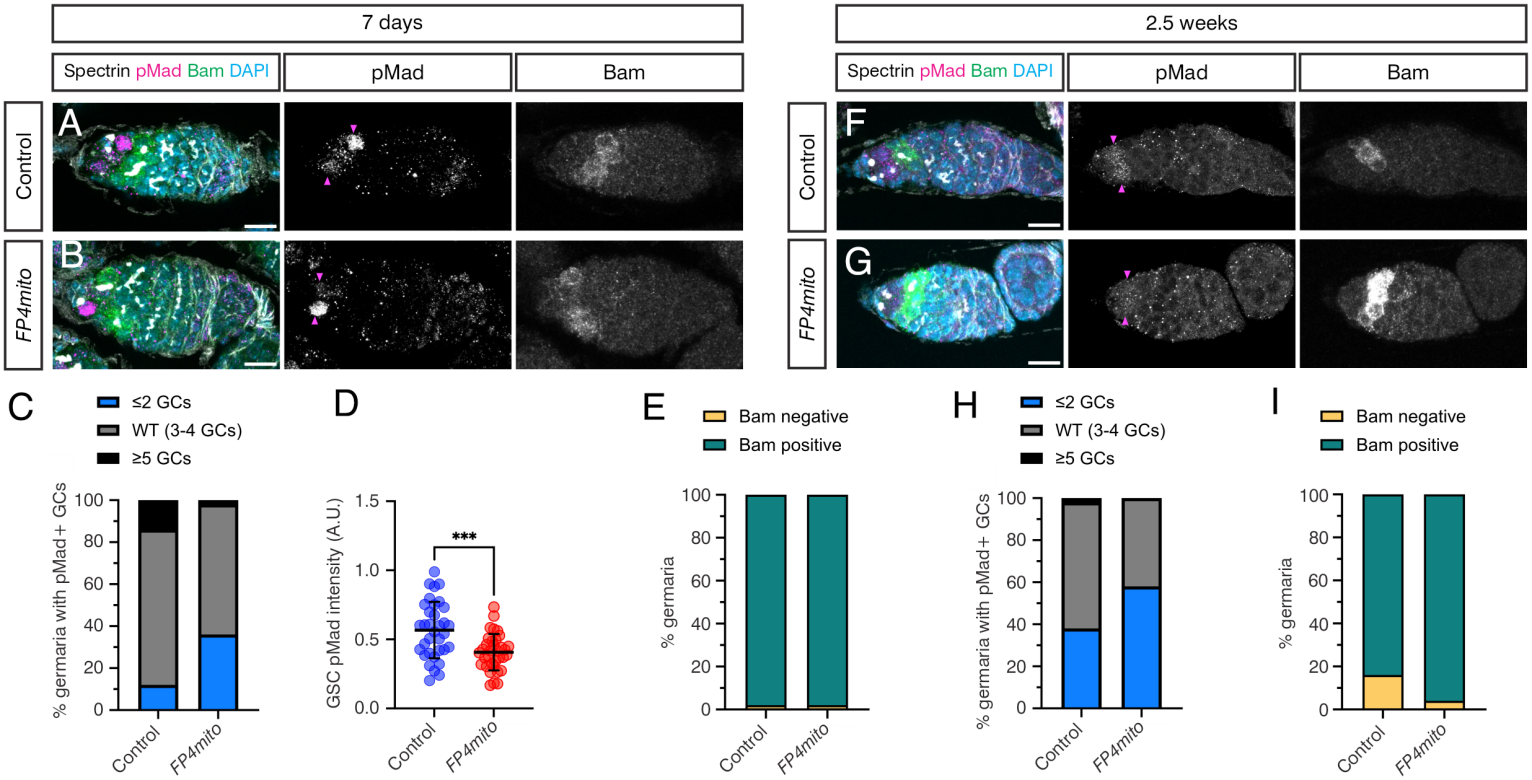
Ena mislocalization impairs BMP signal reception and stem cell fitness. (*A* and *B*) Representative images of control or *FP4mito* germaria aged for 7 d, immunostained with anti-αSpectrin (gray), anti-pMad (magenta and *Inset*), anti-Bam (green and *Inset*), and DAPI (blue). Magenta arrowheads indicate pMad-positive GCs. (Scale bar, 10 μm.) (*C*) Graph shows the number of pMad-positive GCs counted in 7-d-old germaria including the representative germaria shown in (*A* and *B*). n = 50. (*D*) Graph shows the pMad intensity in GSCs in control and *FP4mito* backgrounds. n = 34 per genotype, data shown as mean ± SD, Welch’s two-tailed unpaired *t* test, *P* = 0.0003 for comparisons between genotypes. (*E*) Graph shows the proportion of germaria with and without Bam-positive cysts counted in 7-d-old samples. n = 50. (*F* and *G*) As in (*A* and *B*), except the germaria were aged for 2.5 wk. (*H*) Graph shows the number of pMad-positive GCs counted in 2.5-wk-old germaria, including the representative germaria from (*F* and *G*). n = 50. (*I*) Graph shows the proportion of germaria with and without Bam-positive cysts counted in 2.5-wk-old samples. n = 50.

As cytonemes enable GSCs to access niche-sequestered Dpp, we tested the effect of perturbing cytoneme formation on BMP signaling and stem cell self-renewal by staining for pMad, Bam, Spectrin, and DAPI in *FP4mito* and control germaria. The number of pMad-positive GCs in each background was quantitated. Wildtype germaria typically contain 3 to 4 pMad-positive GCs, i.e., GSCs and CBs, whereas 2 or less indicates aberrant differentiation and 5 or more indicates delayed differentiation (19). By 1-wk posteclosion, there is an increase in the number of pMad-positive GCs lost to differentiation in *FP4mito* germaria compared to controls (Fig. 5 *A*–*C*). Furthermore, GSCs expressing *FP4mito* display significantly reduced pMad levels (Fig. 5*D*). Quantitation also reveals that almost all the germaria contain Bam-positive cells or cysts (Fig. 5*E*), showing that the ability of GCs to dedifferentiate is unperturbed by *FP4mito* expression. Together, these data reveal that there is reduced Dpp signal reception and GSC loss in germaria from 1-wk-old females when cytonemes are disrupted.

By 2.5 wk posteclosion, we observe an increase in the percentage of control and *FP4mito* germaria exhibiting aberrant differentiation (Fig. 5 *F*–*H*), relative to 1-wk posteclosion (Fig. 5*C*), consistent with age-related loss. At 2.5 wk, more *FP4mito* germaria show GSC loss, based on 2 or less pMad-positive cells (Fig. 5*H*). There is also a small proportion of germaria that are Bam-negative, e.g., that contain 16 cc, consistent with age-related decline (Fig. 5*I*). Together, these data show that mislocalization of Ena perturbs cytoneme formation and the ability of GSCs to respond to niche BMP signaling, resulting in a decline in the stem cell pool.

### Ena Mislocalization Impairs GC Dedifferentiation.

Given that perturbation of cytoneme formation disrupts BMP signal reception, we next used *FP4mito* to test whether cytonemes are necessary for GC dedifferentiation. Specifically, we transiently induced the *hs-bam* transgene as before (Fig. 1*A*), in either a *nosGAL4VP16*>*FP4mito* or *nosGAL4VP16* control background. We also performed a control HS experiment with *nosGAL4VP16*>*FP4mito* or *nosGAL4VP16* flies that lacked the *hs-bam* transgene, using the same HS conditions (*SI Appendix*, Fig. S4 *A*–*F*). Quantitation of these germaria at 1, 3, and 6 dpHS reveals that almost all contain pMad-positive GSCs (*SI Appendix*, Fig. S4*G*), although there is some variation in the relative numbers over time and trends toward GSC loss for *FP4mito* germaria (*SI Appendix*, Fig. S4*H*). Therefore, HS in the absence of an *hs-bam* transgene did not induce ectopic GC differentiation in control or *FP4mito* germaria over the time course (*SI Appendix*, Fig. S4*I*). Consistent with this, most germaria have Bam-positive differentiating cells (*SI Appendix*, Fig. S4*J*), showing that HS in the absence of an *hs-bam* transgene is insufficient to induce aberrant GC differentiation.

**Fig. 6.**
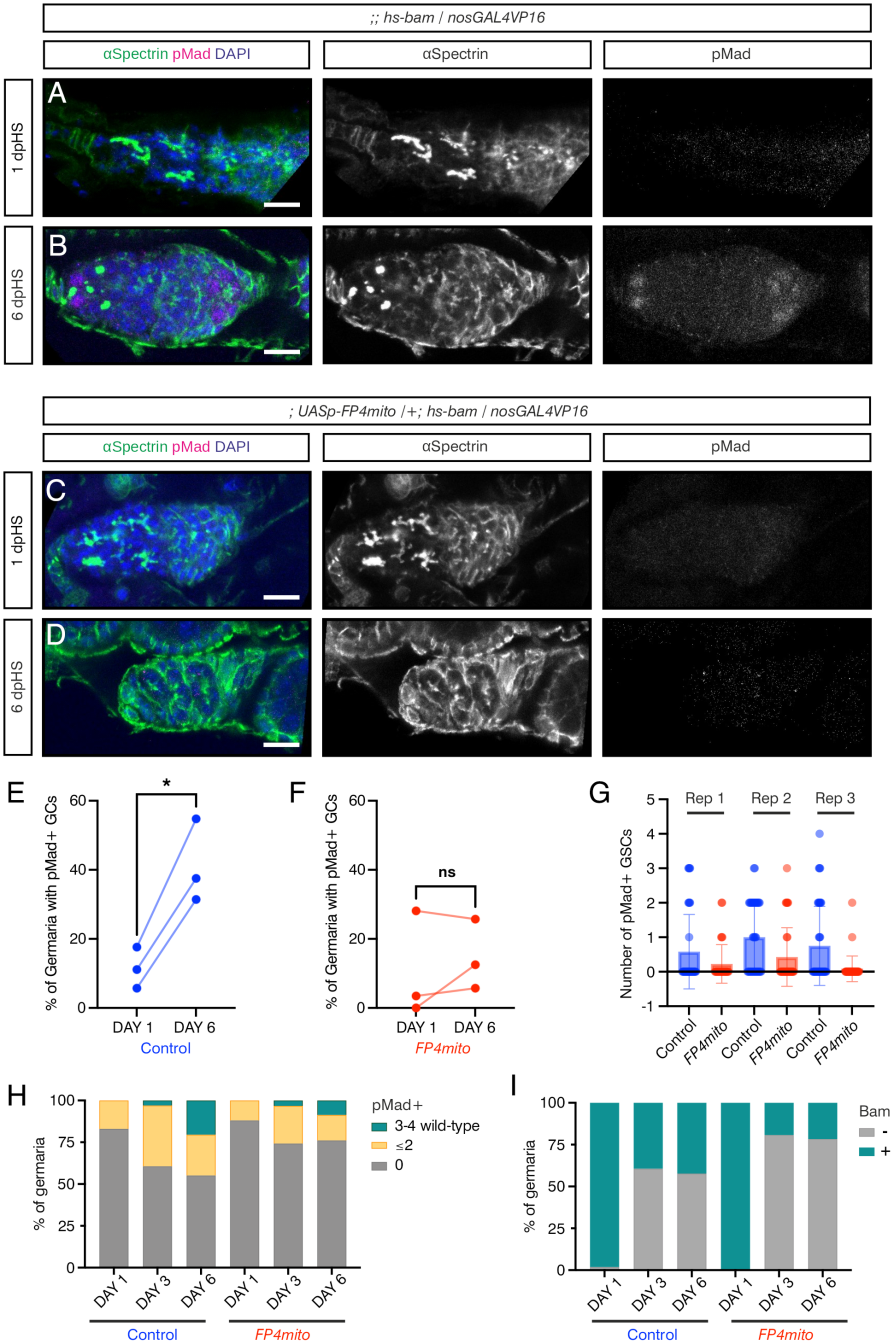
Ena mislocalization impairs GSC dedifferentiation. (*A* and *B*) Control germaria carrying an *hs-bam* transgene and *nosGAL4VP16* immunostained with anti-pMad (magenta and *Inset*), anti-αSpectrin (green and *Inset*), and DAPI (blue). Representative germaria are shown for day 1 (*A*) and 6 (*B*) post-HS. (Scale bar, 10 μm.) (*C* and *D*) As in (*A* and *B*), except germaria also carry a *UASp-FP4mito* transgene. (Scale bar, 10 μm.) (*E* and *F*) Quantification of germaria containing pMad-positive early GCs on days 1 and 6 post-HS for (*E*) control and (*F*) *FP4mito*. Data are pairwise comparisons, two-tailed paired *t* test comparing day 1 to day 6, *P* = 0.0150 for control (*) and *P* = 0.448 for *FP4mito* (ns). Each repeat contains at least 30 germaria. (*G*) Quantification of the number of pMad-positive GSCs per germaria for each repeat. Data are shown as mean ± SD. (*H* and *I*) Graph shows the number of pMad-positive GCs (*H*) or Bam-positive GCs (*I*) counted in 1, 3, and 6 dpHS (representative examples are in *SI Appendix*, Fig. S5 *A*–*J*).

Next, we performed the same experiment in control and *FP4mito* germaria that also carried the *hs-bam* transgene. We performed a pair-wise comparison of the number of germaria with pMad-positive GCs (i.e., GSCs or CBs) 1 vs. 6 dpHS. On day 1 in both conditions, the majority of GCs are pMad negative and have branched fusomes (Fig. 6 *A*, *C*, *E*, and *F*). On day 6, a proportion of control *nosGAL4VP16* germaria contain pMad-positive GSCs and a round spectrosome, consistent with niche recolonization (Fig. 6*B*). Germaria lacking pMad-positive cells and round fusomes were also observed (*SI Appendix*, Fig. S5*A*), indicative of a lack of germline recovery such that all GCs were lost due to differentiation. Quantitation of these data show that ~40% of control germaria have a significant recovery of pMad-positive GCs (Fig. 6*E*), consistent with the data presented in Fig. 1.

*FP4mito* germaria with either pMad-negative (Fig. 6*D*) or pMad-positive GCs (*SI Appendix*, Fig. S5*B*) were also observed 6 dpHS. However, in contrast to control germaria, there is little or no recovery of pMad-positive GSCs in *FP4mito* expressing germaria (Fig. 6*F*, cf Fig. 6*E*). Additionally, control germaria have higher numbers of pMad-positive GSCs compared to *FP4mito* germaria (Fig. 6*G*), indicating that the *FP4mito* GCs are less able to respond to Dpp signaling.

It is possible that less dedifferentiation is observed in *FP4mito* germaria because the cells dedifferentiate normally but are then rapidly lost again to differentiation. To test this, we performed Bam, pMad, Spectrin, and DAPI staining on control and *FP4mito* germaria carrying the *hs-bam* transgene, at 1, 3, and 6 dpHS (*SI Appendix*, Fig. S5 *C*–*L*). Quantitation of the number of pMad-positive cells again reveals lower dedifferentiation to pMad-positive cells in the *FP4mito* germaria (Fig. 6*H* and *SI Appendix*, Fig. S5 *M* and *N*). Between days 3 and 6, the proportion of pMad-positive dedifferentiated cells is constant in either the control or *FP4mito* backgrounds, but the cells become more dedifferentiated over time so that there is an increase in the relative proportion of germaria with 3 to 4 pMad-positive cells (Fig. 6*H*) and GSCs (*SI Appendix*, Fig. S5*O*). These results are inconsistent with dedifferentiation occurring normally in the presence of *FP4mito*, followed by rapid GSC loss, which would result in a reduction in the number of pMad-positive cells at day 6 relative to day 3, rather than an increase.

Similarly, quantitation of Bam-positive cells over the time course reveals that the proportion of differentiating cells is unchanged between days 3 and 6 for each of the control or *FP4mito* backgrounds (Fig. 6*I*). The GCs that do not dedifferentiate are Bam-negative, a characteristic of later-stage cysts, consistent with the GCs remaining differentiated. Together, these results indicate that FP4mito, which causes Ena mislocalization and subsequent cytoneme dysfunction, prevents cyst dedifferentiation. These data are consistent with cytonemes playing an important role in the ability of cysts to respond to the self-renewal Dpp signal which is necessary for GCs to recolonize an empty niche.

## Discussion

Here, we provide evidence that actin-rich cytonemes, which are present on all early ovarian GCs (Figs. 2 *E* and *F*, 3 *B*–*G*, and 4) (19), are necessary for robust BMP signal activation and niche occupancy during both homeostasis and dedifferentiation. Our data also show that Ena loss-of-function can be a useful and targeted tool for dysregulating cytoneme function in different contexts. Ena is localized to cytoneme tips, as observed in the filopodia of leading-edge cells during dorsal closure (46). Using a *FP4mito* transgene to mislocalize Ena resulted in less frequent and shorter cytonemes, again consistent with the effects of Ena mislocalization on filopodia (41, 46). While there are contexts where *FP4mito* expression phenocopies *ena* loss-of-function mutants (41, 48), there are also examples where a stronger or different phenotype is observed with *FP4mito* compared to *ena* mutants (46, 48). This is likely because a subset of cellular Diaphanous (Dia), a processive actin polymerase, can be mislocalized to mitochondria with Ena upon expression of *FP4mito* (46, 47). Indeed, loss of Dia disrupts cytoneme formation in ovarian GSCs and other contexts (5, 19, 39, 49, 50), and both Ena and Dia also have roles in mediating adhesion, with Dia having a role in Ecad endocytosis in epithelial cells (51) and GSCs (19). Bam promotes GC differentiation by the posttranscriptional down-regulation of Ecad (52, 53) which must be reexpressed during dedifferentiation. Therefore, it is possible that disruption to adhesion could impact dedifferentiation by precluding the formation of stable GC–niche interactions. However, *FP4mito* expression does not appear to affect GSC–niche Ecad levels at homeostasis (Fig. 3 *J* and *K*), suggesting that Dia function is largely unaffected.

**Fig. 7.**
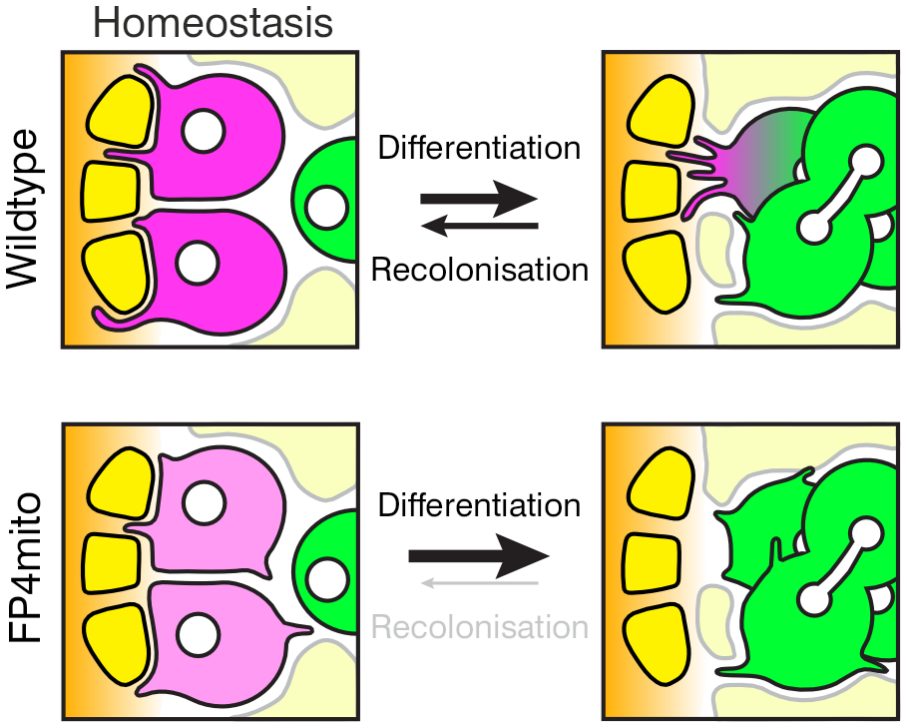
Cytonemes promote stem cell fitness and enable cyst dedifferentiation. Model of the role of cytonemes in the ovarian germline at homeostasis, in promoting Dpp signaling and stem cell fitness, and in dedifferentiation, by enabling GCs to access niche-derived Dpp.

We show that cytonemes coordinate niche occupancy and responsiveness to the BMP self-renewal signal during homeostasis and dedifferentiation (Fig. 7). While it would have been interesting to study physiological dedifferentiation, this is a very rare event that occurs at a frequency of only 0.2% (54). Therefore, we relied on inducing a transient *hs-bam* pulse, which is the standard assay in the field for studying dedifferentiation (35, 36, 54). We have also established a tissue culture-based assay for studying BMP cytonemes, as we show that BMP2 beads can induce contacts by Tkv-loaded cytonemes from S2R+ cells (Fig. 2 *K*–*N*). A similar approach with ESCs and Wnt beads has revealed new insights into cytoneme-Wnt source contacts, including similarities with glutamatergic neuronal synapses (6). An S2 cell model for studying Hh-containing cytonemes revealed that Dispatched stabilizes cytonemes and was predictive of their in vivo functionality in the *Drosophila* wing disc (40). Therefore, the S2R+ cell cytoneme-BMP bead model will be useful in future studies for dissecting mechanisms and making predictions that can be tested in ovarian GCs and other contexts.

Our findings demonstrate a requirement for cytonemes to promote stem cell fitness. While the cytoneme regulators we targeted by knockdown in our previous study affected either cytoneme number or length (19), an advantage of targeting Ena is that both are reduced (Fig. 4). Mislocalizing Ena reveals that GSCs with disrupted cytonemes have lower BMP signaling and reduced niche occupancy (Figs. 5 and 7). Cytonemes also direct contact with the BMP source during recolonization of the niche (Figs. 6 and 7), and previous studies have shown how cytonemes can orient toward and be stabilized by Dpp-expressing cells (37–39). Moreover, our in vitro experiments demonstrate that a BMP source is sufficient to attract and/or stabilize cytonemes, consistent with cytonemes facilitating selective and stable niche interactions. Therefore, our data support cytonemes playing a fundamental role in establishing/maintaining niche interactions and asymmetric signaling, which are central to maintaining the stem cell fate.

Although CBs extend cytonemes in all directions during germline homeostasis, the cytonemes are longer and more oriented toward niche cells during dedifferentiation (Fig. 2 *E*–*I*), which likely reflects in part the space available for cytoneme extension. This cytoneme polarity allows the differentiating cells to become responsive to the self-renewal Dpp signal again, facilitating niche recolonization and polarized signaling. This is similar to the interplay between cytonemes and stemness during ovarian GSC maintenance, whereby cytonemes allow receipt of the Dpp signal from niche cells, which then promotes the stem cell fate. In further feedback regulation, Dpp signaling induces a second type of GSC cytoneme to establish a Dpp signaling threshold, which allows the daughter that exits the niche to differentiate (19). Similar interdependent relationships between the cause and effect of cytonemes in mediating asymmetric signaling and niche organization have been described in other contexts. For example, *Drosophila* AMPs extend polarized cytonemes to contact FGF-expressing wing disc niche cells at adherens junctions. This anchors FGF receptor-loaded AMP cytonemes to niche cells, resulting in cytoneme-dependent asymmetric FGF signaling, which reinforces AMP polarity, niche-proximal position, and stemness (5). Additionally, *Drosophila* testis GSCs extend cytoneme-like structures called microtubule-dependent nanotubes (MT-nanotubes) into the niche to collect the self-renewal Dpp signal, with Dpp required to induce or stabilize MT-nanotubes (55).

Only a proportion of cysts are capable of dedifferentiating (Fig. 1) (54), with 4 cc and 8 cc previously shown to differentiate (35). As we only detect cytonemes up to the 8 cc stage (Fig. 2*B* and *SI Appendix*, Fig. S1*A*), this may explain why dedifferentiation of 16 cc does not occur. Therefore, the presence of cytonemes is likely one factor limiting dedifferentiation. Recently, the changes in the transcriptome and translatome during GSC differentiation have been described, and this has revealed two major waves of changes separated by an inflection point around the 4 cc stage. From the 8 cc stage onward, gene expression changes associated with terminal differentiation occur (56). This transition is also linked to dramatic changes in actin dynamics. Waves of contractile cortical actin are suppressed in 4 to 8 cc and initiate in 16 cc to promote their encapsulation by follicle cells prior to oocyte maturation (57). It is possible that the gene expression and proteome changes that occur in the 8 cc prevent the cells from forming cytonemes, further committing the cells to terminal differentiation.

Our data also show that the presence of extra niche cells is associated with a failure to dedifferentiate (Fig. 2 *B* and *D* and *SI Appendix*, Fig. S1 *A*, *B*, and *G*). The additional somatic cells we observed colonizing the niche are likely to be ECs, which initially enter the empty niche and respond to Dpp (Fig. 2*B*) (36). These data suggest that the ECs colonizing the niche can both form a barrier to GSC–niche contact and outcompete the differentiated cysts for Dpp. Additionally, it is possible that, during homeostasis, niche occupancy by GSCs prevents the CB cytonemes from accessing the niche to ensure that the CBs differentiate rather than dedifferentiate. Based on our findings, we suggest that a general prerequisite for dedifferentiation in diverse contexts is cytoneme-mediated niche contact and that other cells can be a major barrier to achieving this. For example, while *Drosophila* AMPs proximal to the niche extend polarized cytonemes to contact niche cells and receive FGF signals to maintain stemness, more distal AMPs have lateral cytonemes, lose niche contact, and differentiate (5). It is possible that, in this context, the presence of proximal AMPs physically blocks distal cells’ cytonemes from contacting the niche, which contributes to these cells remaining in a more differentiated state.

Some ECs express low levels of Dpp, which can weakly promote germline cell dedifferentiation (54). We suggest that soluble Dpp from ECs may initially be able to weakly aid dedifferentiation, but it is insufficient to promote cytoneme contact and BMP responsiveness. In support of this, our data show that cytonemes are oriented toward the niche rather than ECs during dedifferentiation, even when there are extra niche cells present that blocks dedifferentiation (Fig. 2*B*). Similarly, germline cells are largely insensitive to the EC released Dpp (54) and cells that have exited the niche can derepress *bam* transcription and differentiate. Different possibilities exist for the general insensitivity of germline cells to the Dpp secreted by ECs. First, the Dpp level may be too low. Second, the insensitivity may reflect that ECs lack the heparan sulfate proteoglycan Dally (58, 59), which binds Dpp (60). Previously, we showed that Tkv activation on cytonemes occurs at sites where Dally-Dpp is concentrated, suggesting that Dally may present Dpp to the cytoneme (19). It is possible that in the absence of Dally presentation, cytonemes cannot recognize the soluble Dpp released by ECs. This would be similar to the role of the glycosylphosphatidylinositol (GPI) anchor in presenting the FGF ligand to air sac primordium (ASP) cytonemes for contact-dependent release and signal reception. In contrast, the ASP cytonemes cannot respond to secreted FGF ligand (61). Third, it is possible that the germline cells respond to the EC Dpp in a cytoneme-independent manner resulting in a qualitatively different response to cytoneme-mediated Dpp reception, which cannot support dedifferentiation. Potential support for this comes from data from the male germline showing that soluble Dpp elicits a different signaling response to the contact-dependent response to niche Dpp (62).

Stem cell maintenance and dedifferentiation are critical for tissue damage repair and underpin the regenerative medicine strategies that rely on stem cell treatments or cellular reprogramming to induced pluripotent stem cells (63–65). As such, manipulating cytonemes could improve regenerative medicine outcomes by favoring particular cell fate outcomes. In addition, defective stem cell maintenance and dedifferentiation are associated with diseases, including aging-related diseases (66), neurodevelopmental disorders (64, 67), and different cancer types (68, 69). Therefore, cytonemes may represent a potential therapeutic target for treating the broad spectrum of diseases caused by defective stem cell maintenance and dedifferentiation.

## Materials and Methods

### Fly Stocks.

All crosses were undertaken at 25 °C on standard fly media [yeast 50 g/L, glucose 78 g/L, maize 72 g/L, agar 8 g/L, 10% (v/v) nipagin in ethanol 27 mL/L, and propionic acid 3 mL/L]. Stocks used are listed in *SI Appendix*, Table S1. Transgenic line generation is described in *SI Appendix*, *Materials and Methods*.

### Heat Shock.

Flies 1 to 3 d posteclosion were heat shocked in vials containing Whatman paper to absorb condensation. Vials were heat shocked in a water bath for 1.5 h at 37 °C and then returned to 25 °C for 2 h before a second 1.5 h heat shock at 37 °C. Flies were then transferred to a new vial and returned to 25 °C for the duration of the experiment.

### Immunofluorescence.

For standard antibody stain, ovaries were dissected in PBS and fixed in 4% formaldehyde for 20 min at room temperature. Ovaries were blocked in 5× Western Blocking buffer (Merck) or 10% BSA in PBST (0.1% Tergitol 15-S-9 in PBS) for 1 h and then incubated with primary antibodies (*SI Appendix*, Table S3) at 4 °C overnight. They were then washed in PBSTw (0.1% Tween 20 in PBS) before incubation at room temperature for 2 h in 0.1× WB with secondary antibodies. Phalloidin (0.033 μM) and DAPI staining was carried out in the penultimate wash for 30 min at room temperature, and ovaries were mounted in Prolong Diamond or Gold (Invitrogen).

For staining of cytonemes, ovaries were dissected in PEM buffer (80 mM PIPES, 5 mM EDTA, 1 mM Magnesium chloride, pH7.4 adjusted with NaOH) and fixed immediately in PEM with 4% formaldehyde, 5 μM paclitaxel (Merck), 1 μM calcium chloride for 30 min at room temperature. Washes were carried out in PBST, and ovaries were then processed as above.

To use anti-Bam and anti-αSpectrin together, anti-αSpectrin was labeled with Zenon AlexaFluor 555 (Thermo Fisher Scientific, according to the manufacturer’s instructions) and added postsecondary antibody incubation and washes. Ovaries were incubated with Zenon labeled anti-αSpectrin for 1 h at room temperature.

### Live Imaging.

Live imaging was performed as described (70).

Imaging settings, quantification, and statistical tests are described in *SI Appendix*, *Materials and Methods*.

### S2R+ Cell Culture and Immunofluorescence.

S2R+ cells (DGRC, RRID:CVCL_Z831) were maintained at 25 °C in Schneider’s Drosophila Medium (Gibco) supplemented with 10% fetal bovine serum (FBS; Gibco) and 1% penicillin-streptomycin (Merck) and plated onto poly-L-lysine (Merck) coated coverslips at 0.75 × 10^6^ density. Cells (0.75 × 10^6^) were cotransfected with 200 ng pAc-Tkv-mCherry (Thickveins C-terminal mCherry fusion) and pAc-Mad-FLAG (71) plasmids each using Effectene reagent (Qiagen). After a 72-h incubation, cells were resuspended in serum-free media and plated onto poly-L-lysine (Merck) coated coverslips at a 0.75 × 10^6^ density. They were serum-starved for 1 h before addition of serum-free medium containing 3 × 10^5^ BSA- or BMP2-conjugated beads (see below). The cells were incubated for 3 h at 25 °C before fixation (40). Samples were permeabilized for 1 h at room temperature in 0.1% Igepal CA-630 (Merck), 5% BSA (Merck), 0.3 M glycine (Fisher Scientific, CAS: 56-40-6) in 1× PBS, followed by overnight incubation at 4 °C of primary antibodies in 1% BSA in PBSTw (0.1% Tween 20 in PBS) (*SI Appendix*, Table S3). Cells were washed four times in PBS and incubated for 2 h with secondary antibodies in 1% BSA in PBSTw. Samples were washed twice in PBSTw for 10 min, and DAPI staining was carried out in the penultimate wash. Coverslips were further washed twice in PBSTw before mounting in ProLong Diamond (Invitrogen). Only cells with Tkv-mCherry fluorescence and beads within 50 µm of the nucleus were imaged. The bead to nucleus distance was calculated for all beads close to a cell if the distance was within 50 µm.

### Bead Conjugation.

BSA and Human/Mouse/Rat BMP-2 Recombinant Protein (Gibco, 120-02C-10UG) were immobilized onto 2.8 µm Dynabeads M-270 carboxylic acid (Invitrogen) as previously described (72). Briefly, 30 µg beads were activated by a 30-min incubation with carbodiimide (Merck) and N-hydroxylsuccinamide (Merck). Following activation, beads were washed three times with 25 mM cold 2-(N-morpholino) ethanesulfonic acid (MES; Merck) buffer (pH5), then incubated for 1 h with either 0.1% BSA in PBSTw (0.1% Tween 20 in PBS) or 500 ng BMP2 (Gibco, 120-02C-10UG) in 25 mM MES buffer. The beads were washed with PBS before resuspending them in 1% BSA PBS and stored at 4 °C.

### C2C12 Cell Culture, Immunofluorescence, and pSmad1/5 Quantitation.

C2C12 cells were maintained in DMEM high-glucose media (Sigma) supplemented with 2 mM L-glutamine (Gibco), and 10% FBS (Gibco) at 37 °C. Cells were seeded at a density of 1 × 10^5^ cells/well on coverslips. After 6 h, the cells were serum-starved overnight. The cells were then incubated in serum-free DMEM (Sigma) with 11 nM soluble BMP2 (Gibco, 120-02C-10UG) or 3 × 10^5^ BSA- or BMP2-conjugated beads. Cells were incubated at 37 °C for 2 h before being washed in PBS and fixed with 4% PFA for 20 min. Samples were permeabilized in 0.2 M glycine and 0.5% Triton X-100 in PBS for 30 min and blocked using 2% fish skin gelatin in PBS. Cells were incubated at room temperature with anti-pSmad1/5 (*SI Appendix*, Table S3) for 1 h, washed in PBS, and incubated with secondary antibody for 30 min. Antibodies were diluted in 2% fish skin gelatin in PBS. Samples were washed in PBS and mounted in ProLong Gold with DAPI (ThermoFisher Scientific). pSmad1/5 quantitation was performed as described (73), with an appropriate intensity threshold for each replicate. For the bead experiments, only cells in contact with a bead were included in the quantitation.

## Supplementary Material

Appendix 01 (PDF)

## Data Availability

All study data are included in the article and/or *SI Appendix*.
